# An exploratory study of the perceived impact of health problems of landmine/UXO victims versus another disability group

**DOI:** 10.1186/1477-7525-10-121

**Published:** 2012-09-28

**Authors:** Russell B Wyper

**Affiliations:** 1 Curtin University, GPO box U1987, Perth, Western Australia, 6845, Australia

## Abstract

**Background:**

The purpose of this exploratory study is to pilot a biopsychosocial instrument called the Perceived Impact of Problem Profile (PIPP) on a cohort of landmine/Unexploded Ordnance (UXO) victims with lower limb disability versus a cohort of persons with similar disability due to other trauma or medical causes. The aim is to provide greater understanding of the psychosocial impact of landmine/UXO injury to inform victim assistance ^a^ interventions within Lao PDR.

**Methods:**

This study employs a mixed methods design, which involved piloting the PIPP instrument through an interviewer administered questionnaire and demographic questionnaire. Fifty one participants were interviewed in both urban and rural locations within Lao PDR.

**Results:**

An analysis of the data reveals significant differences in perceived impact for pain, anxiety and how recently the injury/illness occurred. Both groups complained of high levels of anxiety and depression; landmine/UXO victims who complained of anxiety and depression reported a much greater impact on life satisfaction and mood.

**Conclusion:**

The perceived impact of the disability is greatest on psychosocial factors for both cohorts, but especially in landmine/UXO victims emphasising the need to focus on improving psychosocial interventions for landmine/UXO victims within Victim assistance programmes in Lao PDR.

## Introduction

Lao PDR is one of the most heavily UXO contaminated countries in the world [1]. It is a small, landlocked country with a population of 6.8 million and has ‘least developed country’ status according to the United Nations Human Development Index [2]. During the Indochina conflict, Laos was the site of extensive aerial bombings and concentrated ground battles. Throughout the period 1964 to 1973 more than 500,000 bombing missions dropped over two million tonnes of ordnance on the country making it, per capita, the most heavily bombed nation in the world [1]. The failure rate of some weapons systems was as high as 30%, particularly for some cluster munitions (such as BLU 26 sub-munition). Once dropped these act in a similar way to a landmine, activated when moved, disturbed or hit [3].

This study is of importance because of the prevalence of people with landmine/UXO related disability in Lao PDR. A recent in-country survey for example, recorded 50,136 casualties between 1964 and 2008. Of the casualties recorded 20,726 (41.34%) survived with injuries [1].

These victims must be seen in the wider context of disability and estimates by the United Nations Economic and Social Commission for Asia and the Pacific (UNESCAP) suggest that 8% of the national population is likely to be disabled [4]. Many disabled people in Lao PDR, as in most developing countries in the world, live in poverty, have limited opportunities for accessing education, health, suitable housing and employment opportunities [4]. Although there are a number of laws and policies pertaining to people with disabilities, including their right to decent and productive work and access to basic services, most persons with disabilities are handicapped by social, economic, physical and political conditions. This situation is worsened by the stigma of disability, poor understanding of the abilities and aspirations of disabled persons, and lack of rehabilitation services [5].

In Lao PDR mental health services are extremely limited as are psychosocial programmes for all persons with disability. However, it is landmine/UXO victims who seem to be most in need of assistance. The few studies that have been undertaken highlight higher levels of mental illness such as depression, suicidal thoughts and substance abuse among landmine/UXO victims. The literature also outlines the link between poverty and landmine/UXO injury and discusses how injury compounds poverty for the individual and the victim’s family increasing the psychological impact of the injury. This is because the vast majority of contamination is in rural areas creating a significant threat to those who are already among the most vulnerable members of society with a correlation being found between UXO pollution and access to basic public services [6]. With few employment opportunities, villagers living in affected areas often find that they are confronted with "enforced risk-taking." They either continue to live in acute poverty and in many cases chronic malnutrition, or risk injury and death by working UXO-contaminated land [7]. Injured villagers living in acute poverty also have the least access to health care. As a result there is often a delay in reaching treatment during the acute phase of their injury. This delay in treatment may also result in greater chronic disability. Rehabilitation of landmine/UXO victims in Lao PDR focuses predominantly on provision of prostheses and on physical rehabilitation; there are very few psychosocial interventions available to landmine/UXO victims. Researchers emphasise the need for further research to improve understanding of the needs of landmine/UXO victims.

This study looks at whether the Perceived Impact of Problem Profile (PIPP) instrument may be useful in this endeavour as it is an aetiologically neutral biopsychosocial instrument that can be used to assess health and functioning [8]. It is a generic research and clinical measurement tool which uses The International Classification of Functioning, Disability and Health (ICF) approved by the World Health Assembly in 2001 [9] as a reference framework. This instrument was chosen because 1) currently there is no validated instrument available for assessing the psychosocial impact of disability due to landmine/UXO injuries, 2) it is understandable and easy to use requiring less capacity development to implement it, and 3) it has been validated in different linguistic and cultural contexts.

It can help allow understanding of a person's subjective experience, which is essential for accurate diagnosis, improving health outcomes and providing appropriate care [10]. It is also hoped that the development of standardised survey tools will help to ensure that data collection proceeds according to appropriate scientific methods and allows the comparison of data between differing regions and countries [11], because Lao PDR is not alone and globally there is scant information available on the psychosocial impacts of disability following landmine/UXO injury [12]. In one of the few studies that have been undertaken, Gunaratnam and colleagues noted that the psychosocial impact of landmine injuries needed to be considered seriously in rehabilitation work [12].

In summary, by conducting this exploratory study it is hoped that a greater insight will be given into the differences in perception between UXO/landmine victims and people with other disabilities, which could help contribute to future rehabilitation programmes. It will also test the PIPP instrument to ascertain if it is a useful tool for research in this field, which could guide policy not just in Lao PDR but also in other vulnerable regions.

## Methods

This research was undertaken as an exploratory study piloting the PIPP instrument in the setting of Humanitarian Mine Action (HMA) in Lao PDR to assess the perceived impact of health conditions on a cohort of mine/UXO victims and a cohort of persons with mobility impairment of different aetiology to make a comparison to see if mine/UXO victims perceive the impact of their disability differently.

### Design of the study

This is a mixed methods design involving piloting the PIPP instrument through an interviewer administered questionnaire consisting of the PIPP instrument and demographic questionnaire.

### The setting

The study was undertaken in three locations; the National Rehabilitation Centre (NRC) in Vientiane where persons with disability receive prostheses, orthoses and physical rehabilitation services, the research was also undertaken at participants’ homes in rural villages in the two most heavily landmine/UXO contaminated provinces of Xieng Khouang and Savannakhet.

### Type of participants

A total of 51 participants were interviewed for the study. All participants have mobility impairment due to injury or illness affecting the limbs. The participants were for the purposes of the study divided into two categories:

1. Disability due to accident involving landmines or UXO

2. Disability not due to Landmine/UXO (e.g. vehicle accident, logging accident) or due to medical illness (e.g. diabetes, skin ulcers)

### Materials involved

The interviewer-administered questionnaire consisted of two components. The first had demographic questions and questions regarding the person’s injury, illness, cause of disability, level of disability and access to health care. This component of the questionnaire was adapted from Epidemiological Tools and Capability and Social Reintegration Tools, which were developed by Physicians for Human Rights [11]; these surveys are used extensively in HMA surveys. The second component of the questionnaire was the PIPP instrument, which collected data on the perceived impact of the health problem on 23 items which were grouped into five domains of psychology, mobility, relationships, self care and participation.

The questionnaire was administered in face to face interviews in the local language by experienced medical researchers from the University of Health Sciences in Vientiane.

### Type of analysis

Quantitative data was obtained using the PIPP instrument, which measures the degree of perceived impact ranging from no impact to extreme impact. This is treated as ordinal data in the subsequent analysis.

Calculation of the raw scores for the five subscales was conducted as follows:

### Raw score calculation

The procedure described in Table 1 below adjusts the score for each subscale according to the number of items in the subscale. The final adjusted subscale score is therefore converted back to the original scoring used for each item (from 1 = no impact, to 6 = extreme impact). If the person has answered all items, with no ticks in the not applicable section, the scoring is:

**Table 1:** Raw Score Calculation for PIPP instrument

**Subscale**	**Step 1**	**Possible range (raw)**	**Step 2**	**Possible range (adjusted)**
Psychological	Add items 1 to 5	5 to 30	Divide score by 5	1 to 6
Self Care	Add items 6 to 9	4 to 24	Divide score by 4	1 to 6
Mobility	Add items 10 to 14	5 to 30	Divide score by 5	1 to 6
Participation	Add items 15 to 19	5 to 30	Divide score by 5	1 to 6
Relationships	Add items 20 to 23	4 to 24	Divide score by 4	1 to 6

If a person ticks the not applicable box for any of the items then their total score for that subscale was divided by the number of items they actually answered, rather than the number listed in Step 2 above. For this research study the scoring system above was used.

It is also recommended to use raw scores when the sample size is less than 100; and for sample sizes greater than 100, Rasch analysis ^b^ is recommended as a check on validity and reliability however the sample size of this study is 51; therefore Rasch analysis was not used [13].

All data was cleaned and entered into SPSS version 17.

## Results

### Causation and medical profile of the sample

The demographic profile of participants was taken and can be seen in Table 2. Questions relating to the cause of disability, use of mobility equipment and medical complaints are summarised in Table 3 below.

**Table 2:** Demographic Profile of Participants

**Demographic**	**Representation**		
**Age**	**Range 19 – 82 years**		
	**Mean 49 years**		
**Gender**	**Total sample**	**Male 71%**	**Female 29%**
	**Landmine/UXO**	**Male 85%**	**Female 15%**
	**Non-UXO**	**Male 54%**	**Female 46%**
**Relationships**	**Married**	**78%**	
	**Defacto**	**2%**	
	**Separated**	**10%**	
	**Never Married**	**10%**	
**Educational Status**	**No formal education**	**36%**	
	**Primary level**	**29%**	
	**Secondary level**	**29%**	
	**Tertiary level**	**6%**	
**Ethnicity**	**Lao Lung**	**71%**	
	**Lao Theung**	**10%**	
	**Lao Sung**	**10%**	
	**Other**	**9%**	
**Religion**	**Buddhist**	**76%**	
	**Christian**	**2%**	
	**Worship Spiritual Ghosts**	**22%**	
**% Head of Household total sample**	**60%**		
**Average number of children per household**	**2**		
**Average number of adults per household**	**3**		

**Table 3:** Causation and Medical Profile of Sample


**Cause of loss of mobility total sample**	**Male**	**+Medical 14%**	**Trauma (non-UXO) 22%**	**Trauma (UXO) 64%**
	**Female**	**Medical 60%**	**Trauma (non-UXO) 13%**	**Trauma (UXO) 27%**
**Injury Cause in landmine/UXO group**	**Antipersonnel landmine**		**34%**	
	**UXO**		**32%**	
	**Unknown**		**17%**	
**Upper limb injury in addition lower limb injury**	**14% males, 7% females**			
**Activity at time of injury in Landmine/UXO sample**	**Walking**		**17%**	
	**Farming/Herding**		**12%**	
	**Travelling**		**11%**	
	**Collecting firewood**		**9%**	
	**Burning rubbish**		**6%**	
	**Forestry**		**6%**	
	**Scrap metal collection**		**6%**	
	**Fishing/hunting**		**3%**	
	**Demining**		**3%**	
	**Other/no response**		**27%**	
**Use of mobility equipment**	**Prostheses**		**Landmine/UXO 74%**	**Non-UXO 50%**
	**Wheelchair**		**Landmine/UXO 0%**	**Non-UXO 16%**
	**Crutches**		**Landmine/UXO 21%**	**Non-UXO 15%**
	**None**		**Landmine/UXO 5%**	**Non-UXO 19%**
**Complained of anxiety/depression since injury or illness**	**Landmine/UXO**		**Yes 83%**	**No 17%**
	**Non-UXO**		**Yes 79%**	**No 21%**
**Complained of chronic pain since injury or illness**	**Landmine/UXO**		**Yes 63%**	**No 37%**
	**Non-UXO**		**Yes 46%**	**No 54%**
**Had access to medical care following accident/illness**	**Landmine/UXO**		**Yes 78%**	**No 22%**
	**Non-UXO**		**Yes 58%**	**No 42%**

### Results of perceived impact of injury between groups ( Additional file 1: Annex 1)

Additional file 1: Annex 1 presents the results of the Mann Whitney ^c^ tests for the demographic and injury variables for the sample population. Although no strict hypothesis was formulated prior to the study, it was assumed that certain differences in perceived impact would exist between various groups. In particular it was assumed that perceived impact would be higher for UXO victims than the Non-UXO group, females over males, and those without prostheses over those with. To test this, variables were grouped to determine if any significant differences exist in terms of their perceived impact on the various PIPP questions.

The most significant differences in PIPP responses were instead a result of employment status, those who felt they were treated differently, those that have someone to care for them, those with upper limb injuries and those that complain of anxiety and depression ( Additional file 1: Annex 1).

There was no is no significant difference between UXO and Non-UXO victims in the PIPP scores.

### Results of perceived impact of injury between UXO and Non-UXO victims ( Additional file 1: Annex 2)

Additional file 1: Annex 2 shows the comparison of UXO and non-UXO cohorts when the data is split between the two and the Mann Whitney test is re-run.

UXO victims reported a higher median impact than non-UXO victims in the psychology domain. They also reported a higher impact on independence and reliance on others. In individual PIPP items relating to self-care UXO victims reported a greater impact and this is especially true of those who have upper limb amputations.

### Comparison of individual PIPP items ( Additional file 1: Annex 3)

An analysis was undertaken of different variables to see if there is a significant difference in the perceived impact within the PIPP items, for example do you suffer from anxiety (yes or no). The comparison was made on the 23 PIPP questions of those who reported anxiety and those that did not. The results can be seen in Additional file 1: Annex 3.

PIPP scores were not affected by physical rehabilitation or prostheses. Specific PIPP scores were mostly affected by limb injury (self-care and personal relationships) and paraplegia (personal relationships). More broadly PIPP scores were affected by those who were treated differently and those suffering from anxiety or depression and pain amongst UXO victims. Time elapsed since injury was a strong contributing factor to the perceived impact on many of the PIPP domains.

### The Spearman’s Correlation ( Additional file 1: Annex 4)

Results of the Spearman’s correlation ^d^ are shown in Additional file 1: Annex 4, the significant results are summarised as follows:

Spearman’s confirms results from Mann Whitney that there is no correlation between receipt of physical therapy/rehabilitation and perceived quality of life (PIPP scores)

Being currently employed is negatively correlated with year of injury, so the more recent the injury the less likely to be currently employed, this is significant at the .01 level

The more recent the injury the higher the perceived distress level in the psychology raw score.

The same goes for participation and relationship scores, the more recent the injury the higher the perceived impact.

## Discussion

Although there is no significant difference between UXO and Non-UXO victims in the PIPP scores ( Additional file 1: Annex 1), when splitting the data between UXO and non-UXO and re-running the Mann Whitney significant differences do emerge ( Additional file 1: Annex 2). Anxiety or Depression, Life Satisfaction and Psychology, and Pain showed the greatest areas where differences arise. Equally interesting were the other factors on the perceived impact of health problems that emerged in the results such as Time Elapsed After Injury. These results highlight areas that should be given extra attention in the future.

### Significant differences between landmine/UXO victims versus another disability group

#### Anxiety or depression

80% of the total sample complained of anxiety or depression since the accident or illness. 82% of landmine/UXO victims reported episodes of anxiety and depressions since the accident and 79% of the Non-UXO cohort reported anxiety and depression since the accident or illness.

A comparison between landmine/UXO victims and non-UXO victims for those respondents that reported suffering from anxiety and depression reveal very interesting results. Landmine/UXO victims report a significantly higher impact on life satisfaction, mood, confidence, independence and reliance on others than the non-UXO cohort (p < .05). The impact on mobility and some items of self care was significantly higher than the non-UXO cohort as was the ability to form close personal relationships and on maintaining relationships with family and friends ( Additional file 1: Annex 1).

#### Life satisfaction and psychology

Both UXO and non-UXO cohorts reported very high median impact on life satisfaction (5 out of 6), mood (4 out of 6) and confidence (4 out of 6) caused by their disability. UXO victims reported a higher median impact than non-UXO victims on the psychology domain. UXO victims also reported a higher impact on independence and reliance on others. This suggests that there is a significant requirement for psychosocial support for both cohorts but particularly UXO victims as a priority.

#### Pain

Impact on life satisfaction and mood in the total sample complaining of pain is counter-intuitive; there is no significant difference on perceived impact on life satisfaction and mood of those complaining of pain in the total sample; however, when those complaining of pain are separated into landmine/UXO victims and non UXO significant differences emerge. Landmine/UXO victims reported significantly higher impact on life satisfaction, moods and feelings, self care, participation in family activities, relationships with relatives and ability to work.

### Other significant factors in perceived impact of health problems

#### Time elapsed since injury

Sixty nine percent of all respondents reported that their injury occurred before 2005. Time elapsed since injury is a strong contributing factor to the perceived impact on many of the PIPP domains. Those who experienced the illness or accident more recently (within the last five years) scored significantly worse on confidence, independence, mobility and participation in community and family activities were compared to those who were injured after 2005. The impact on the psychology domain is significant in this group; this may support the need for early psychosocial intervention following the accident or illness.

### Factors that did not have an impact

#### Gender

Research exploring gender and disability indicates the disadvantageous and marginalised situation for women with disabilities, with researchers observing women with disabilities as often experiencing a 'double' disadvantage due to the combined effects of gender and disability [14]. Findings suggest that women with a disability, as compared with men with a disability and women without a disability, faced a double disadvantage in terms of employment, education, and income levels, and were much less likely to marry, and more likely to become divorced if married prior to onset of the disability. Women with disabilities are likely to be poorer, less healthy and more socially isolated than their male counterparts [15]; this was not evident in this data in terms or perceived impact. There was no significant difference between the genders in the PIPP scores.

#### Prostheses/physical rehabilitation

Interestingly there was no significant difference in perceived impact between those who receive prostheses and physical rehabilitation and those who did not, supported by Mann Whitney and Spearman’s correlation tests. Current interventions for persons with disability in Lao PDR focus largely on physical rehabilitation rather than psychosocial interventions. This could imply that the participant has had the prosthesis for a long time and therefore is no longer aware of its impact, or that the participants may not be using the prosthesis effectively. However, further investigation needs to be conducted to explore reasons why there is no significant difference in perceived impact.

#### Interpretation

The sample size of the experiment was too small to conclusively give a profile of difference between landmine/UXO victims and other disabled groups. However enough differences emerged between the groups when assessing the individual items to support further investigation in certain areas and generally the need for psychosocial interventions for persons with disability especially landmine/UXO victims.

The PIPP instrument could be used to further understand the impact on individuals of disabling injuries and could be incorporated into future research. The PIPP tool could also improve the communication between landmine/UXO victims and care givers, which may lead to improved care and empowerment of the victim.

#### Study limitations

This is an exploratory non-random study to explore the use of the PIPP tool within Humanitarian Mine Action and the sample size is small, therefore results are not representative of the landmine/UXO population. Also, due to the sample size, Rasch analysis was not undertaken as a check on validity and reliability. The developers of the PIPP instrument recommend using Rasch analysis for sample sizes greater than 100. For sample sizes less than 100 however, they recommend summing raw scores into 5 subscales/domains (psychology, self care, mobility, participation and relationships). Following this methodology, there was no significant differences between the UXO and non UXO groups; however analysis of the individual PIPP items demonstrated significant differences for some demographic and injury variables. Summing the raw scores into the 5 subscales may mask significant results, therefore, when conducting similar research in samples of less than 100 it may be advisable to also analyse the individual PIPP items.

#### Suggestions for further research

The PIPP tool could be considered as one tool to be used in conjunction with other survey instruments such as the SF-36 ^e^ instrument and the Health Assessment questionnaire ^f^ to improve understanding of the impact of landmine/UXO injury. The limitations of medical and rehabilitation services do not make it feasible to use the PIPP instrument to tailor intervention to the needs of the individual when landmine/UXO victims do not have access to even basic services let alone a multi-faceted health care service which incorporates medical care, prosthetic services, rehabilitative services, psychosocial services, economic reintegration etc. However, use of the PIPP tool may be used to improve understanding of the needs of landmine/UXO victims which then may facilitate improved VA programme planning and development of more holistic interventions as more resources become available both within Lao PDR and internationally. The dearth of research into the psychosocial impact of landmine/UXO injury needs to be addressed as does the lack of psychosocial interventions in Lao PDR. In the immediate term combining current resources and improving coordination of landmine/UXO interventions may provide an immediate solution until resources can be mobilised and capacities improved. One solution for Lao PDR may be to enhance the physical rehabilitation outreach programmes currently undertaken by NRC/COPE and develop or incorporate a psychosocial capacity within these teams either by developing the capacity of the physical rehabilitation staff or by partnering with a Non-Governmental Organization (NGO) such as BasicNeeds to provide a psychosocial capacity within the rehabilitation team. Any intervention will need to build on current indigenous capacities and be implemented within the context of cultural and religious beliefs and practises using a community based approach that is accessible to all persons with disability. This is but one of many options available; the NRA Victim assistance technical working group is currently investigating various options. The international community should support initiatives to improve psychosocial research and interventions in Lao PDR and a full support package should be developed for all persons with disability and their families especially landmine/UXO victims.

## Conclusion

Despite the limitations of the study due to sample size, this was a successful exploration into perceived impact of health problems amongst landmine victims and other disabled groups. The PIPP instrument will be a useful tool in future research. Areas that emerge as warranting further research especially amongst UXO/Landmine survivors are anxiety or depression, life satisfaction and psychology and pain. Equally time elapsed since injury should be looked into for all groups as results showed it to be very significant.

## Endnotes

^a^**Victim assistance** – Victim assistance includes, but is not limited to, casualty data collection, emergency and continuing medical care, physical rehabilitation, psychological support and social reintegration, economic reintegration, and laws and public policies to ensure the full and equal integration and participation of survivors, their families and communities in society. Taken from Landmine Monitor Report 2010. International Campaign to Ban landmines.

^b^Rasch analysis is a statistical measurement method that allows the measurement of an attribute independently of particular tests or indices. It creates a linear representation using many individual items, ranked by item difficulty. A well performing Rasch model will have items hierarchically placed from simple to more difficult, and individuals with high abilities should be able to perform all the items below a level of difficulty. The Rasch model is statistically strong because it enables ordinal measures to be converted into meaningful interval measures. It also allows information from various tests or tools with different scoring systems to be applied using the Rasch model.

^c^Mann–Whitney *U* test is a nonparametric statistical test to compare two groups.

^d^Spearman’s correlation: a shortcut formula for *r* when you have two sets of ranks.

^e^SF-36: The Short Form (36) Health Survey is a survey of patient health. The SF-36 is a measure of health status and is commonly used in health economics as a variable in the quality-adjusted life year calculation to determine the cost-effectiveness of a health treatment. The original SF-36 came out from the Medical Outcome Study, MOS, done by the RAND Corporation.

^f^Health Assessment Questionnaire: The Health Assessment Questionnaire (HAQ) was originally developed in 1978 by James F. Fries, MD, and colleagues at Stanford University. It was one of the first self-report functional status (disability) measures and has become the dominant instrument in many disease areas.

## Abbreviations

HMA: Humanitarian Mine Action; ICF: International Classification of Functioning, Disability and Health; NGO: Non-Governmental Organization; NRA: National Regulatory Authority (Lao PDR); NRC: National Rehabilitation Centre; PDR: People’s Democratic Republic; PIPP: Perceived Impact of Problem Profile; PHR: Physicians for Human Rights; UNDP: United Nations Development Programme; UNESCAP: United Nations Economic and Social Commission for Asia and the Pacific; UXO: Unexploded Ordnance; WHO: World Health Organisation.

## Competing interests

The author declares no competing interests.

## Author contributions

RW carried out the research for this paper and drafted the manuscript. The author read and approved the final manuscript.

## Supplementary Material

Additional file 1 **Annex 1.** Results of Perceived Impact of Injury between groups. **Annex 2.** Results of Perceived Impact of Injury between UXO and Non UXO victims. **Annex 3.** Mann–Whitney Hypothesis Testing UXO versus Non-UXO. **Annex 4.** Spearman’s Correlations. Click here for file
