# Supplementary material for: An exploratory study of the perceived impact of health problems of landmine/UXO victims versus another disability group
Source: Health Qual Life Outcomes. 2012 Sep 28;10:121. doi: 10.1186/1477-7525-10-121 (PMC3493282; doi:10.1186/1477-7525-10-121)
Supplement: Additional file 1 — Annex 1. Results of Perceived Impact of Injury between groups. Annex 2. Results of Perceived Impact of Injury between UXO and Non UXO victims. Annex 3. Mann–Whitney Hypothesis Testing UXO versus Non-UXO. Annex 4. Spearman’s Correlations. [file 1477-7525-10-121-S1.doc]

Annex 1

**Table 3** **Results of Perceived Impact of Injury between groups**

|  | Head of Household | | | | | | | Limb Injury/Non Amp | | | | | | | | | Paraplegic | | | | | | | | | | | | Receive Physical Therapy or Rehab | | | | |
| --- | --- | --- | --- | --- | --- | --- | --- | --- | --- | --- | --- | --- | --- | --- | --- | --- | --- | --- | --- | --- | --- | --- | --- | --- | --- | --- | --- | --- | --- | --- | --- | --- | --- |
|  | Mann–Whitney U | | | Asymp. Sig. | | | | Mann–Whitney U | | | | Asymp. Sig. | | | | | Mann–Whitney U | | | | | | | Asymp. Sig. | | | | | Mann–Whitney U | | | | Asymp. Sig. |
| Your overall satisfaction with life | 263.00 | | | 0.34 | | | | ***208.50*** | | | | ***0.02*** | | | | | 174.50 | | | | | | | 0.71 | | | | | 223.50 | | | | 0.43 |
| Your moods and feelings | 291.00 | | | 0.71 | | | | ***184.50*** | | | | ***0.01*** | | | | | 163.00 | | | | | | | 0.51 | | | | | 183.50 | | | | 0.10 |
| Your sense of confidence | 289.00 | | | 0.68 | | | | 311.50 | | | | 0.84 | | | | | 150.00 | | | | | | | 0.32 | | | | | ***119.50*** | | | | ***0.00*** |
| Your ability to live independently | 265.50 | | | 0.38 | | | | ***197.50*** | | | | ***0.02*** | | | | | 165.00 | | | | | | | 0.54 | | | | | 216.50 | | | | 0.36 |
| Your reliance on others for help | 243.50 | | | 0.19 | | | | ***174.00*** | | | | ***0.00*** | | | | | 168.00 | | | | | | | 0.59 | | | | | 229.00 | | | | 0.52 |
| Your ability to wash yourself | 253.50 | | | 0.24 | | | | ***152.00*** | | | | ***0.00*** | | | | | 116.00 | | | | | | | 0.05 | | | | | 255.50 | | | | 0.94 |
| Your ability to use the toilet | 278.00 | | | 0.52 | | | | ***172.00*** | | | | ***0.00*** | | | | | 131.50 | | | | | | | 0.14 | | | | | 247.00 | | | | 0.79 |
| Your ability to dress yourself | 273.50 | | | 0.45 | | | | ***146.00*** | | | | ***0.00*** | | | | | ***95.00*** | | | | | | | ***0.01*** | | | | | 248.00 | | | | 0.80 |
| Your ability to feed yourself | 289.00 | | | 0.63 | | | | ***173.50*** | | | | ***0.00*** | | | | | ***93.00*** | | | | | | | ***0.00*** | | | | | 229.00 | | | | 0.45 |
| Your ability to sit or stand | 258.50 | | | 0.31 | | | | ***185.00*** | | | | ***0.01*** | | | | | 144.50 | | | | | | | 0.26 | | | | | 235.00 | | | | 0.61 |
| Your ability to carry things | 225.00 | | | 0.09 | | | | 298.50 | | | | 0.65 | | | | | 174.00 | | | | | | | 0.70 | | | | | 177.50 | | | | 0.08 |
| Your ability to use a vehicle | 242.00 | | | 0.15 | | | | 247.00 | | | | 0.12 | | | | | 187.50 | | | | | | | 0.97 | | | | | 256.50 | | | | 0.95 |
| Your ability to move around within your house | 288.50 | | | 0.66 | | | | 251.00 | | | | 0.16 | | | | | 151.00 | | | | | | | 0.33 | | | | | 226.00 | | | | 0.47 |
| Your ability to move around your neighbourhood | 271.50 | | | 0.44 | | | | 292.50 | | | | 0.57 | | | | | 189.00 | | | | | | | 1.00 | | | | | ***142.50*** | | | | ***0.01*** |
| Your ability to participate in family activities | 248.00 | | | 0.22 | | | | 280.50 | | | | 0.42 | | | | | 164.50 | | | | | | | 0.54 | | | | | 231.50 | | | | 0.55 |
| Your ability to assist other members of your family | 280.00 | | | 0.55 | | | | 276.50 | | | | 0.38 | | | | | 170.00 | | | | | | | 0.63 | | | | | 209.50 | | | | 0.28 |
| Your ability to participate in community activities | 286.50 | | | 0.64 | | | | 295.00 | | | | 0.60 | | | | | 169.00 | | | | | | | 0.61 | | | | | 233.50 | | | | 0.58 |
| Your ability to participate in activities that you enjoy | 265.50 | | | 0.38 | | | | 294.50 | | | | 0.59 | | | | | 175.00 | | | | | | | 0.72 | | | | | 242.50 | | | | 0.72 |
| Your ability to work | 306.50 | | | 0.94 | | | | 269.00 | | | | 0.29 | | | | | 160.00 | | | | | | | 0.45 | | | | | 243.00 | | | | 0.72 |
| Your ability to have a close relationship with others | 281.50 | | | 0.56 | | | | ***194.00*** | | | | ***0.01*** | | | | | ***69.00*** | | | | | | | ***0.00*** | | | | | 250.50 | | | | 0.85 |
| Your ability to relate to neighbours and friends | 271.00 | | | 0.44 | | | | 319.50 | | | | 0.96 | | | | | ***114.00*** | | | | | | | ***0.06*** | | | | | ***168.00*** | | | | ***0.05*** |
| Your ability to relate to relatives | 264.50 | | | 0.36 | | | | 311.50 | | | | 0.84 | | | | | ***107.50*** | | | | | | | ***0.04*** | | | | | ***181.00*** | | | | ***0.09*** |
| Your ability to relate to people in authority | ***203.00*** | | | ***0.03*** | | | | 239.50 | | | | 0.11 | | | | | 144.00 | | | | | | | 0.25 | | | | | 194.00 | | | | 0.16 |
| **Raw Subscale Scores** |  | | |  | | | |  | | | |  | | | | |  | | | | | | |  | | | | |  | | | |  |
| **Psychology** | 263.50 | | | 0.37 | | | | ***180.50*** | | | | ***0.01*** | | | | | 165.50 | | | | | | | 0.56 | | | | | 186.50 | | | | 0.13 |
| **Self Care** | 261.00 | | | 0.33 | | | | ***129.00*** | | | | ***0.00*** | | | | | ***104.50*** | | | | | | | ***0.03*** | | | | | 249.50 | | | | 0.84 |
| **Mobility** | 310.00 | | | 1.00 | | | | 243.00 | | | | 0.13 | | | | | 183.50 | | | | | | | 0.89 | | | | | 184.50 | | | | 0.12 |
| **Participation** | 273.00 | | | 0.47 | | | | 283.00 | | | | 0.46 | | | | | 184.50 | | | | | | | 0.91 | | | | | 238.50 | | | | 0.66 |
| **Relationships** | 231.00 | | | 0.12 | | | | 289.50 | | | | 0.53 | | | | | 138.50 | | | | | | | 0.21 | | | | | 180.50 | | | | 0.09 |
|  | Prostheses Receipt | | | | | | | Employed before Injury | | | | | | | | Currently Employed | | | | | | | | | Outreach Programmes in community | | | | | | | | |
|  | Mann–Whitney U | | | Asymp. Sig. | | | | Mann–Whitney U | | | | Asymp. Sig. | | | | Mann–Whitney U | | | | Asymp. Sig. | | | | | Mann–Whitney U | | | | | | | | Asymp. Sig. |
| Your overall satisfaction with life | 188.50 | | | 0.49 | | | | 152.50 | | | | 0.35 | | | | 221.50 | | | | 0.07 | | | | | 260.50 | | | | | | | | 0.84 |
| Your moods and feelings | 196.50 | | | 0.63 | | | | ***91.50*** | | | | ***0.01*** | | | | 283.00 | | | | 0.59 | | | | | 257.00 | | | | | | | | 0.78 |
| Your sense of confidence | 216.00 | | | 1.00 | | | | 167.50 | | | | 0.59 | | | | 217.00 | | | | 0.07 | | | | | 236.50 | | | | | | | | 0.48 |
| Your ability to live independently | 168.00 | | | 0.24 | | | | 136.50 | | | | 0.18 | | | | 243.50 | | | | 0.19 | | | | | 225.50 | | | | | | | | 0.35 |
| Your reliance on others for help | 177.50 | | | 0.35 | | | | 145.00 | | | | 0.26 | | | | 257.50 | | | | 0.30 | | | | | 207.50 | | | | | | | | 0.18 |
| Your ability to wash yourself | 205.00 | | | 0.78 | | | | ***103.00*** | | | | ***0.02*** | | | | 281.50 | | | | 0.56 | | | | | 229.00 | | | | | | | | 0.36 |
| Your ability to use the toilet | 183.00 | | | 0.41 | | | | ***111.50*** | | | | ***0.04*** | | | | 260.50 | | | | 0.32 | | | | | 256.50 | | | | | | | | 0.77 |
| Your ability to dress yourself | 203.50 | | | 0.75 | | | | ***103.00*** | | | | ***0.02*** | | | | 297.00 | | | | 0.79 | | | | | 247.00 | | | | | | | | 0.61 |
| Your ability to feed yourself | 195.50 | | | 0.56 | | | | 159.50 | | | | 0.38 | | | | 284.00 | | | | 0.55 | | | | | 239.50 | | | | | | | | 0.45 |
| Your ability to sit or stand | 198.50 | | | 0.67 | | | | ***101.50*** | | | | ***0.03*** | | | | 272.50 | | | | 0.46 | | | | | 247.50 | | | | | | | | 0.64 |
| Your ability to carry things | 196.50 | | | 0.63 | | | | 125.00 | | | | 0.11 | | | | 229.00 | | | | 0.11 | | | | | 248.00 | | | | | | | | 0.64 |
| Your ability to use a vehicle | 154.00 | | | 0.11 | | | | 172.50 | | | | 0.66 | | | | ***216.00*** | | | | ***0.05*** | | | | | 266.50 | | | | | | | | 0.94 |
| Your ability to move around within your house | 193.50 | | | 0.58 | | | | 155.00 | | | | 0.38 | | | | ***212.50*** | | | | ***0.05*** | | | | | 246.50 | | | | | | | | 0.61 |
| Your ability to move around your neighbourhood | 170.00 | | | 0.26 | | | | 162.00 | | | | 0.49 | | | | ***156.00*** | | | | ***0.00*** | | | | | 223.50 | | | | | | | | 0.32 |
| Your ability to participate in family activities | 178.00 | | | 0.36 | | | | 175.50 | | | | 0.73 | | | | ***179.50*** | | | | ***0.01*** | | | | | 231.50 | | | | | | | | 0.42 |
| Your ability to assist other members of your family | 150.50 | | | 0.11 | | | | 130.50 | | | | 0.14 | | | | ***189.00*** | | | | ***0.02*** | | | | | 237.00 | | | | | | | | 0.48 |
| Your ability to participate in community activities | 165.00 | | | 0.21 | | | | 166.50 | | | | 0.57 | | | | ***153.50*** | | | | ***0.00*** | | | | | 241.50 | | | | | | | | 0.54 |
| Your ability to participate in activities that you enjoy | 182.50 | | | 0.41 | | | | 157.50 | | | | 0.42 | | | | ***188.00*** | | | | ***0.02*** | | | | | 219.00 | | | | | | | | 0.28 |
| Your ability to work | 209.50 | | | 0.87 | | | | 115.50 | | | | 0.06 | | | | ***143.50*** | | | | ***0.00*** | | | | | 242.00 | | | | | | | | 0.54 |
| Your ability to have a close relationship with others | 191.00 | | | 0.53 | | | | 155.50 | | | | 0.38 | | | | 296.00 | | | | 0.78 | | | | | 262.50 | | | | | | | | 0.87 |
| Your ability to relate to neighbours and friends | 189.50 | | | 0.51 | | | | 179.50 | | | | 0.81 | | | | 260.00 | | | | 0.32 | | | | | 249.50 | | | | | | | | 0.66 |
| Your ability to relate to relatives | 185.50 | | | 0.45 | | | | 158.50 | | | | 0.43 | | | | 282.50 | | | | 0.58 | | | | | 266.50 | | | | | | | | 0.94 |
| Your ability to relate to people in authority | 166.00 | | | 0.22 | | | | 148.00 | | | | 0.30 | | | | ***176.00*** | | | | ***0.01*** | | | | | 203.00 | | | | | | | | 0.16 |
| **Raw Subscale Scores** |  | | |  | | | |  | | | |  | | | |  | | | |  | | | | |  | | | | | | | |  |
| **Psychology** | 189.50 | | | 0.53 | | | | 120.50 | | | | 0.09 | | | | 237.50 | | | | 0.16 | | | | | 229.00 | | | | | | | | 0.40 |
| **Self Care** | 199.50 | | | 0.69 | | | | ***101.50*** | | | | ***0.03*** | | | | 288.50 | | | | 0.67 | | | | | 238.00 | | | | | | | | 0.49 |
| **Mobility** | 171.00 | | | 0.28 | | | | 130.50 | | | | 0.15 | | | | ***190.00*** | | | | ***0.02*** | | | | | 235.50 | | | | | | | | 0.47 |
| **Participation** | 166.50 | | | 0.24 | | | | 169.50 | | | | 0.63 | | | | ***151.00*** | | | | ***0.00*** | | | | | 220.50 | | | | | | | | 0.30 |
| **Relationships** | 180.00 | | | 0.38 | | | | 185.50 | | | | 0.93 | | | | ***206.50*** | | | | ***0.04*** | | | | | 232.50 | | | | | | | | 0.43 |
|  | | | Treated differently | | | | | | Injury affected work, learn | | | | | | | | | UXO | | | | | | | | | Injury in the last 4 years | | | | | | |
|  | | | Mann–Whitney | | | Asymp. Sig. | | | Mann–Whitney | | | | Asymp. Sig. | | | | | Mann–Whitney | | | | Asymp. Sig. | | | | | Mann–Whitney | | | Asymp. Sig. | | | |
| Your overall satisfaction with life | | | ***153.50*** | | | ***0.02*** | | | 45.00 | | | | 0.07 | | | | | 306.00 | | | | 0.72 | | | | | 230.00 | | | 0.29 | | | |
| Your moods and feelings | | | ***144.50*** | | | ***0.01*** | | | 89.50 | | | | 0.87 | | | | | 313.50 | | | | 0.84 | | | | | 207.00 | | | 0.13 | | | |
| Your sense of confidence | | | 223.00 | | | 0.39 | | | 73.50 | | | | 0.46 | | | | | 278.00 | | | | 0.37 | | | | | ***160.50*** | | | ***0.01*** | | | |
| Your ability to live independently | | | ***94.50*** | | | ***0.00*** | | | 78.50 | | | | 0.58 | | | | | 283.50 | | | | 0.43 | | | | | ***181.00*** | | | ***0.04*** | | | |
| Your reliance on others for help | | | ***95.50*** | | | ***0.00*** | | | 54.00 | | | | 0.15 | | | | | 262.50 | | | | 0.23 | | | | | 213.00 | | | 0.16 | | | |
| Your ability to wash yourself | | | ***100.00*** | | | ***0.00*** | | | 74.00 | | | | 0.45 | | | | | 252.00 | | | | 0.14 | | | | | 228.00 | | | 0.26 | | | |
| Your ability to use the toilet | | | ***99.50*** | | | ***0.00*** | | | 68.50 | | | | 0.35 | | | | | 296.50 | | | | 0.59 | | | | | 226.50 | | | 0.25 | | | |
| Your ability to dress yourself | | | ***100.00*** | | | ***0.00*** | | | 71.00 | | | | 0.39 | | | | | 249.00 | | | | 0.13 | | | | | 232.50 | | | 0.30 | | | |
| Your ability to feed yourself | | | ***165.50*** | | | ***0.01*** | | | 83.50 | | | | 0.66 | | | | | 247.50 | | | | 0.08 | | | | | 279.50 | | | 0.99 | | | |
| Your ability to sit or stand | | | ***169.00*** | | | ***0.04*** | | | 94.00 | | | | 1.00 | | | | | 281.00 | | | | 0.41 | | | | | 250.50 | | | 0.54 | | | |
| Your ability to carry things | | | 188.50 | | | 0.11 | | | 70.00 | | | | 0.39 | | | | | 241.50 | | | | 0.11 | | | | | 207.00 | | | 0.13 | | | |
| Your ability to use a vehicle | | | 187.50 | | | 0.09 | | | 46.00 | | | | 0.07 | | | | | 232.50 | | | | 0.06 | | | | | 228.50 | | | 0.26 | | | |
| Your ability to move around within your house | | | ***123.50*** | | | ***0.00*** | | | 77.50 | | | | 0.55 | | | | | 305.50 | | | | 0.72 | | | | | ***146.50*** | | | ***0.00*** | | | |
| Your ability to move around your neighbourhood | | | 208.00 | | | 0.24 | | | 64.50 | | | | 0.29 | | | | | 260.50 | | | | 0.22 | | | | | ***166.50*** | | | ***0.02*** | | | |
| Your ability to participate in family activities | | | ***168.00*** | | | ***0.04*** | | | 75.50 | | | | 0.51 | | | | | 281.00 | | | | 0.41 | | | | | ***129.50*** | | | ***0.00*** | | | |
| Your ability to assist other members of your family | | | 192.00 | | | 0.13 | | | 86.00 | | | | 0.77 | | | | | 271.00 | | | | 0.31 | | | | | ***166.50*** | | | ***0.02*** | | | |
| Your ability to participate in community activities | | | ***171.00*** | | | ***0.05*** | | | 86.50 | | | | 0.79 | | | | | 285.00 | | | | 0.45 | | | | | ***112.00*** | | | ***0.00*** | | | |
| Your ability to participate in activities that you enjoy | | | 210.50 | | | 0.26 | | | 79.50 | | | | 0.60 | | | | | 285.00 | | | | 0.45 | | | | | ***100.50*** | | | ***0.00*** | | | |
| Your ability to work | | | 186.00 | | | 0.09 | | | 70.50 | | | | 0.39 | | | | | 279.00 | | | | 0.37 | | | | | ***152.00*** | | | ***0.01*** | | | |
| Your ability to have a close relationship with others | | | ***117.00*** | | | ***0.00*** | | | 86.00 | | | | 0.77 | | | | | 244.50 | | | | 0.11 | | | | | 264.00 | | | 0.73 | | | |
| Your ability to relate to neighbours and friends | | | 210.00 | | | 0.25 | | | 71.50 | | | | 0.42 | | | | | 306.00 | | | | 0.73 | | | | | 250.00 | | | 0.53 | | | |
| Your ability to relate to relatives | | | 228.50 | | | 0.45 | | | 64.00 | | | | 0.27 | | | | | 298.50 | | | | 0.62 | | | | | 244.50 | | | 0.45 | | | |
| Your ability to relate to people in authority | | | 241.50 | | | 0.65 | | | 86.00 | | | | 0.77 | | | | | ***195.50*** | | | | ***0.01*** | | | | | ***82.00*** | | | ***0.00*** | | | |
| **Raw Subscale Scores** | | |  | | |  | | |  | | | |  | | | | |  | | | |  | | | | |  | | |  | | | |
| **Psychology** | | | ***101.50*** | | | ***0.00*** | | | 70.00 | | | | 0.40 | | | | | 288.00 | | | | 0.50 | | | | | ***182.00*** | | | ***0.05*** | | | |
| **Self Care** | | | ***84.50*** | | | ***0.00*** | | | 67.00 | | | | 0.33 | | | | | 251.50 | | | | 0.16 | | | | | 239.00 | | | 0.39 | | | |
| **Mobility** | | | ***150.00*** | | | ***0.02*** | | | 70.50 | | | | 0.41 | | | | | 312.00 | | | | 0.82 | | | | | 196.00 | | | 0.09 | | | |
| **Participation** | | | 176.50 | | | 0.07 | | | 82.00 | | | | 0.67 | | | | | 272.50 | | | | 0.33 | | | | | ***102.50*** | | | ***0.00*** | | | |
| **Relationships** | | | 195.00 | | | 0.15 | | | 76.00 | | | | 0.52 | | | | | 245.00 | | | | 0.13 | | | | | ***161.00*** | | | ***0.01*** | | | |
|  | | Marriage | | | | | | | | Someone to Care For You | | | | | | | | | Upper Limb Injury | | | | | | | | | Feel Pain | | | | | |
|  | | Mann–Whitney | | | | | Asymp. Sig. | | | Mann–Whitney | | | | Asymp. Sig. | | | | | Mann–Whitney | | | | Asymp. Sig. | | | | | Mann–Whitney | | | Asymp. Sig. | | |
| Your overall satisfaction with life | | 178.50 | | | | | 0.51 | | | 119.00 | | | | 0.32 | | | | | 100.00 | | | | 0.28 | | | | | 249.50 | | | 0.15 | | |
| Your moods and feelings | | 171.00 | | | | | 0.41 | | | 87.50 | | | | 0.06 | | | | | 101.50 | | | | 0.32 | | | | | 239.00 | | | 0.11 | | |
| Your sense of confidence | | 170.50 | | | | | 0.40 | | | 139.00 | | | | 0.67 | | | | | 127.00 | | | | 0.81 | | | | | 312.00 | | | 0.85 | | |
| Your ability to live independently | | 152.00 | | | | | 0.20 | | | 117.50 | | | | 0.31 | | | | | 98.50 | | | | 0.27 | | | | | 264.50 | | | 0.26 | | |
| Your reliance on others for help | | 147.50 | | | | | 0.16 | | | 120.50 | | | | 0.35 | | | | | 85.50 | | | | 0.14 | | | | | 236.50 | | | 0.10 | | |
| Your ability to wash yourself | | 202.00 | | | | | 0.94 | | | 116.00 | | | | 0.26 | | | | | ***68.50*** | | | | ***0.04*** | | | | | 268.00 | | | 0.27 | | |
| Your ability to use the toilet | | 175.50 | | | | | 0.46 | | | 110.50 | | | | 0.21 | | | | | ***68.00*** | | | | ***0.04*** | | | | | 254.50 | | | 0.18 | | |
| Your ability to dress yourself | | 194.50 | | | | | 0.79 | | | 96.00 | | | | 0.09 | | | | | 79.00 | | | | 0.08 | | | | | 263.50 | | | 0.24 | | |
| Your ability to feed yourself | | 198.50 | | | | | 0.85 | | | 118.00 | | | | 0.23 | | | | | 124.00 | | | | 0.70 | | | | | 319.50 | | | 0.95 | | |
| Your ability to sit or stand | | 198.00 | | | | | 0.87 | | | 129.00 | | | | 0.49 | | | | | 88.00 | | | | 0.16 | | | | | ***213.00*** | | | ***0.04*** | | |
| Your ability to carry things | | 195.50 | | | | | 0.82 | | | 91.50 | | | | 0.08 | | | | | 106.00 | | | | 0.39 | | | | | ***212.00*** | | | ***0.03*** | | |
| Your ability to use a vehicle | | 192.00 | | | | | 0.74 | | | 136.00 | | | | 0.59 | | | | | 122.00 | | | | 0.68 | | | | | 297.00 | | | 0.61 | | |
| Your ability to move around within your house | | 195.00 | | | | | 0.80 | | | 112.00 | | | | 0.23 | | | | | 132.00 | | | | 0.93 | | | | | 302.50 | | | 0.70 | | |
| Your ability to move around your neighbourhood | | 143.50 | | | | | 0.13 | | | ***79.00*** | | | | ***0.03*** | | | | | 108.00 | | | | 0.42 | | | | | 310.50 | | | 0.82 | | |
| Your ability to participate in family activities | | 144.50 | | | | | 0.14 | | | 97.50 | | | | 0.11 | | | | | 119.00 | | | | 0.63 | | | | | 239.50 | | | 0.11 | | |
| Your ability to assist other members of your family | | 204.00 | | | | | 0.98 | | | ***71.50*** | | | | ***0.02*** | | | | | 118.50 | | | | 0.62 | | | | | 225.00 | | | 0.06 | | |
| Your ability to participate in community activities | | 196.00 | | | | | 0.83 | | | 100.00 | | | | 0.13 | | | | | 123.00 | | | | 0.72 | | | | | 276.00 | | | 0.37 | | |
| Your ability to participate in activities that you enjoy | | 201.50 | | | | | 0.93 | | | 98.00 | | | | 0.11 | | | | | 123.00 | | | | 0.72 | | | | | 269.00 | | | 0.30 | | |
| Your ability to work | | 188.00 | | | | | 0.67 | | | 94.00 | | | | 0.09 | | | | | 129.50 | | | | 0.87 | | | | | 262.50 | | | 0.24 | | |
| Your ability to have a close relationship with others | | 203.50 | | | | | 0.97 | | | 129.50 | | | | 0.48 | | | | | 130.50 | | | | 0.89 | | | | | 297.00 | | | 0.62 | | |
| Your ability to relate to neighbours and friends | | 185.50 | | | | | 0.63 | | | 112.00 | | | | 0.24 | | | | | 118.50 | | | | 0.62 | | | | | 284.00 | | | 0.46 | | |
| Your ability to relate to relatives | | 197.50 | | | | | 0.85 | | | 90.50 | | | | 0.07 | | | | | 122.50 | | | | 0.70 | | | | | 299.00 | | | 0.65 | | |
| Your ability to relate to people in authority | | 187.00 | | | | | 0.66 | | | 140.00 | | | | 0.69 | | | | | 116.50 | | | | 0.58 | | | | | 314.50 | | | 0.88 | | |
| **Raw Subscale Scores** | |  | | | | |  | | |  | | | |  | | | | |  | | | |  | | | | |  | | |  | | |
| **Psychology** | | 154.50 | | | | | 0.23 | | | 111.00 | | | | 0.24 | | | | | 95.50 | | | | 0.25 | | | | | 253.00 | | | 0.19 | | |
| **Self Care** | | 202.50 | | | | | 0.95 | | | 106.00 | | | | 0.17 | | | | | ***56.50*** | | | | ***0.02*** | | | | | 260.50 | | | 0.23 | | |
| **Mobility** | | 171.00 | | | | | 0.42 | | | 91.50 | | | | 0.09 | | | | | 112.50 | | | | 0.51 | | | | | 227.00 | | | 0.07 | | |
| **Participation** | | 187.50 | | | | | 0.68 | | | ***70.00*** | | | | ***0.02*** | | | | | 121.00 | | | | 0.68 | | | | | 237.50 | | | 0.11 | | |
| **Relationships** | | 191.50 | | | | | 0.75 | | | 114.50 | | | | 0.27 | | | | | 124.00 | | | | 0.74 | | | | | 312.50 | | | 0.86 | | |
|  | | | | | ANXIETY | | | | | | | | | | Age <50 | | | | | | | | | | | Gender | | | | | | | |
|  | | | | | Mann–Whitney U | | | | | | Asymp. Sig. | | | | Mann–Whitney U | | | | | | Asymp. Sig. | | | | | Mann–Whitney U | | | | | | Asymp. Sig. | |
| Your overall satisfaction with life | | | | | ***70.00*** | | | | | | ***0.00*** | | | | **217.50** | | | | | | **0.04** | | | | | 262.00 | | | | | | 0.86 | |
| Your moods and feelings | | | | | ***107.00*** | | | | | | ***0.02*** | | | | 264.00 | | | | | | 0.29 | | | | | 267.50 | | | | | | 0.96 | |
| Your sense of confidence | | | | | 150.50 | | | | | | 0.19 | | | | 265.50 | | | | | | 0.30 | | | | | 228.00 | | | | | | 0.37 | |
| Your ability to live independently | | | | | ***113.50*** | | | | | | ***0.03*** | | | | 272.50 | | | | | | 0.36 | | | | | 264.00 | | | | | | 0.90 | |
| Your reliance on others for help | | | | | ***84.00*** | | | | | | ***0.00*** | | | | 248.50 | | | | | | 0.17 | | | | | 256.50 | | | | | | 0.77 | |
| Your ability to wash yourself | | | | | 161.00 | | | | | | 0.26 | | | | 318.00 | | | | | | 0.98 | | | | | 263.50 | | | | | | 0.89 | |
| Your ability to use the toilet | | | | | 139.00 | | | | | | 0.10 | | | | 284.00 | | | | | | 0.48 | | | | | 253.50 | | | | | | 0.72 | |
| Your ability to dress yourself | | | | | 138.00 | | | | | | 0.09 | | | | 287.00 | | | | | | 0.51 | | | | | 263.50 | | | | | | 0.89 | |
| Your ability to feed yourself | | | | | 190.50 | | | | | | 0.68 | | | | 312.50 | | | | | | 0.88 | | | | | 268.00 | | | | | | 0.96 | |
| Your ability to sit or stand | | | | | ***95.00*** | | | | | | ***0.01*** | | | | 312.50 | | | | | | 0.90 | | | | | 207.50 | | | | | | 0.19 | |
| Your ability to carry things | | | | | ***81.00*** | | | | | | ***0.00*** | | | | 228.50 | | | | | | 0.08 | | | | | 226.00 | | | | | | 0.35 | |
| Your ability to use a vehicle | | | | | 202.00 | | | | | | 0.94 | | | | 297.50 | | | | | | 0.66 | | | | | 221.00 | | | | | | 0.27 | |
| Your ability to move around within your house | | | | | 136.00 | | | | | | 0.09 | | | | 256.00 | | | | | | 0.21 | | | | | 213.50 | | | | | | 0.22 | |
| Your ability to move around your neighbourhood | | | | | ***125.00*** | | | | | | ***0.05*** | | | | 310.50 | | | | | | 0.87 | | | | | 212.00 | | | | | | 0.22 | |
| Your ability to participate in family activities | | | | | 186.00 | | | | | | 0.64 | | | | 221.50 | | | | | | 0.06 | | | | | 195.00 | | | | | | 0.11 | |
| Your ability to assist other members of your family | | | | | ***104.50*** | | | | | | ***0.01*** | | | | **203.50** | | | | | | **0.02** | | | | | 253.00 | | | | | | 0.72 | |
| Your ability to participate in community activities | | | | | 165.50 | | | | | | 0.33 | | | | 269.00 | | | | | | 0.33 | | | | | 210.50 | | | | | | 0.20 | |
| Your ability to participate in activities that you enjoy | | | | | 178.00 | | | | | | 0.51 | | | | 234.50 | | | | | | 0.10 | | | | | 224.50 | | | | | | 0.33 | |
| Your ability to work | | | | | 165.00 | | | | | | 0.32 | | | | 297.00 | | | | | | 0.66 | | | | | 265.00 | | | | | | 0.91 | |
| Your ability to have a close relationship with others | | | | | 159.00 | | | | | | 0.25 | | | | 303.00 | | | | | | 0.75 | | | | | 235.00 | | | | | | 0.45 | |
| Your ability to relate to neighbours and friends | | | | | 166.50 | | | | | | 0.35 | | | | 262.50 | | | | | | 0.27 | | | | | 229.50 | | | | | | 0.39 | |
| Your ability to relate to relatives | | | | | 157.00 | | | | | | 0.24 | | | | 312.50 | | | | | | 0.90 | | | | | 240.50 | | | | | | 0.53 | |
| Your ability to relate to people in authority | | | | | 192.00 | | | | | | 0.75 | | | | 313.00 | | | | | | 0.91 | | | | | 183.00 | | | | | | 0.07 | |
| **Raw Subscale Scores** | | | | |  | | | | | |  | | | |  | | | | | |  | | | | |  | | | | | |  | |
| **Psychology** | | | | | ***72.50*** | | | | | | ***0.00*** | | | | 249.00 | | | | | | 0.18 | | | | | 268.00 | | | | | | 0.97 | |
| **Self Care** | | | | | 138.50 | | | | | | 0.10 | | | | 294.00 | | | | | | 0.62 | | | | | 245.50 | | | | | | 0.60 | |
| **Mobility** | | | | | ***94.00*** | | | | | | ***0.01*** | | | | 309.50 | | | | | | 0.86 | | | | | 253.00 | | | | | | 0.72 | |
| **Participation** | | | | | 153.00 | | | | | | 0.22 | | | | 238.00 | | | | | | 0.12 | | | | | 224.00 | | | | | | 0.34 | |
| **Relationships** | | | | | 169.00 | | | | | | 0.39 | | | | 307.00 | | | | | | 0.82 | | | | | 215.00 | | | | | | 0.25 | |

Annex 2

**Table 4** **Results of Perceived Impact of Injury between UXO and Non UXO victims**

|  | Non UXO (N=24) | | | UXO (N=27) | | | Total (N=51) | | |
| --- | --- | --- | --- | --- | --- | --- | --- | --- | --- |
|  | Mean | Std.Deviation | Median | Mean | Std. Deviation | Median | Mean | Std. Deviation | Median |
| Pain | 0.46 | 0.51 | 0 | 0.63 | 0.49 | 1 | 0.55 | 0.50 | 1 |
| Anxiety | 0.79 | 0.41 | 1 | 0.81 | 0.40 | 1 | 0.80 | 0.40 | 1 |
| Your overall satisfaction with life | 4.88 | 1.19 | 5 | 4.52 | 1.67 | 5 | 4.69 | 1.46 | 5 |
| Your moods and feelings | 3.88 | 1.65 | 4.5 | 4.04 | 1.63 | 4 | 3.96 | 1.62 | 4 |
| Your sense of confidence | 4.08 | 1.32 | 4 | 3.74 | 1.40 | 4 | 3.90 | 1.36 | 4 |
| Your ability to live independently | 3.33 | 2.08 | 3 | 3.85 | 1.92 | 4 | 3.61 | 1.99 | 3 |
| Your reliance on others for help | 3.17 | 2.06 | 2 | 3.89 | 1.95 | 4 | 3.55 | 2.01 | 3 |
| Your ability to wash yourself | 2.08 | 1.84 | 1 | 2.48 | 1.72 | 2 | 2.29 | 1.77 | 2 |
| Your ability to use the toilet | 2.42 | 1.84 | 2 | 2.63 | 1.88 | 2 | 2.53 | 1.85 | 2 |
| Your ability to dress yourself | 2.00 | 1.67 | 1 | 2.44 | 1.65 | 2 | 2.24 | 1.66 | 2 |
| Your ability to feed yourself | 1.38 | 1.06 | 1 | 1.67 | 1.00 | 1 | 1.53 | 1.03 | 1 |
| Your ability to sit or stand | 3.42 | 2.02 | 3 | 3.93 | 1.71 | 4 | 3.69 | 1.86 | 4 |
| Your ability to carry things | 4.42 | 1.56 | 4.5 | 3.74 | 1.63 | 4 | 4.06 | 1.62 | 4 |
| Your ability to use a vehicle | 3.71 | 2.26 | 4 | 4.81 | 1.80 | 6 | 4.29 | 2.08 | 6 |
| Your ability to move around within your house | 2.71 | 1.81 | 2 | 2.41 | 1.53 | 2 | 2.55 | 1.65 | 2 |
| Your ability to move around your neighbourhood | 3.54 | 1.86 | 4 | 2.85 | 1.81 | 2 | 3.18 | 1.85 | 2 |
| Your ability to participate in family activities | 3.50 | 1.67 | 3 | 3.19 | 1.86 | 2 | 3.33 | 1.76 | 3 |
| Your ability to assist other members of your family | 3.71 | 1.68 | 4 | 3.22 | 1.80 | 2 | 3.45 | 1.75 | 3 |
| Your ability to participate in community activities | 4.25 | 1.80 | 5 | 3.85 | 1.88 | 4 | 4.04 | 1.83 | 5 |
| Your ability to participate in activities that you enjoy | 4.21 | 1.79 | 5 | 3.81 | 1.84 | 4 | 4.00 | 1.81 | 4 |
| Your ability to work | 4.96 | 1.27 | 5.5 | 4.56 | 1.63 | 5 | 4.75 | 1.47 | 5 |
| Your ability to have a close relationship with another person | 2.08 | 1.50 | 1 | 2.67 | 1.80 | 2 | 2.39 | 1.67 | 2 |
| Your ability to relate to neighbours and friends | 2.88 | 1.68 | 2 | 2.78 | 1.91 | 2 | 2.82 | 1.79 | 2 |
| Your ability to relate to relatives | 2.54 | 1.35 | 2 | 2.63 | 1.86 | 2 | 2.59 | 1.63 | 2 |
| Your ability to relate to people in authority | 4.38 | 1.47 | 4 | 3.26 | 1.79 | 2 | 3.78 | 1.72 | 4 |
| **Raw Subscale Scores** |  |  |  |  |  |  |  |  |  |
| Psychology | 3.87 | 1.23 | 3.70 | 4.01 | 1.47 | 4.40 | 3.94 | 1.35 | 4.00 |
| Self Care | 1.97 | 1.46 | 1.38 | 2.31 | 1.44 | 2.00 | 2.15 | 1.45 | 2.00 |
| Mobility | 3.56 | 1.37 | 3.00 | 3.55 | 1.33 | 3.20 | 3.55 | 1.33 | 3.20 |
| Participation | 4.13 | 1.27 | 4.20 | 3.73 | 1.61 | 3.40 | 3.91 | 1.46 | 3.80 |
| Relationships | 2.97 | 1.08 | 2.63 | 2.83 | 1.61 | 2.00 | 2.90 | 1.37 | 2.25 |

Annex 3

**Table 5** **Mann–Whitney Hypothesis Testing UXO versus Non-UXO**

|  | ANXIETY | | | | | | | | | PAIN | | | | | | | | |
| --- | --- | --- | --- | --- | --- | --- | --- | --- | --- | --- | --- | --- | --- | --- | --- | --- | --- | --- |
|  | Non UXO | | | | UXO | | | | | Non UXO | | | | | UXO | | | |
|  | Mann–Whitney | | Asymp. Sig. | | Mann–Whitney | | | Asymp. Sig. | | Mann–Whitney | | Asymp. Sig. | | | Mann–Whitney | | | Asymp. Sig. |
| Your overall satisfaction with life | 32.00 | | 0.23 | | ***2.00*** | | | ***0.00*** | | 71.00 | | 0.97 | | | ***47.50*** | | | ***0.05*** |
| Your moods and feelings | 40.00 | | 0.58 | | ***11.50*** | | | ***0.00*** | | 61.00 | | 0.53 | | | ***32.50*** | | | ***0.01*** |
| Your sense of confidence | 36.00 | | 0.40 | | ***13.00*** | | | ***0.01*** | | 63.00 | | 0.61 | | | 67.50 | | | 0.37 |
| Your ability to live independently | 45.00 | | 0.85 | | ***10.50*** | | | ***0.00*** | | 65.00 | | 0.70 | | | 59.00 | | | 0.18 |
| Your reliance on others for help | 33.50 | | 0.30 | | ***10.50*** | | | ***0.00*** | | 55.50 | | 0.34 | | | 57.50 | | | 0.16 |
| Your ability to wash yourself | 47.50 | | 1.00 | | 30.00 | | | 0.10 | | 61.50 | | 0.51 | | | ***45.00*** | | | ***0.03*** |
| Your ability to use the toilet | 39.50 | | 0.55 | | 29.00 | | | 0.09 | | 70.50 | | 0.95 | | | ***49.00*** | | | ***0.06*** |
| Your ability to dress yourself | 46.50 | | 0.94 | | ***20.00*** | | | ***0.02*** | | 62.50 | | 0.56 | | | ***45.00*** | | | ***0.03*** |
| Your ability to feed yourself | 37.00 | | 0.29 | | 37.00 | | | 0.21 | | 55.50 | | 0.19 | | | ***73.00*** | | | ***0.50*** |
| Your ability to sit or stand | 27.00 | | 0.14 | | ***19.50*** | | | ***0.02*** | | 64.00 | | 0.66 | | | ***37.00*** | | | ***0.01*** |
| Your ability to carry things | 27.50 | | 0.14 | | ***10.50*** | | | ***0.00*** | | 60.50 | | 0.51 | | | ***29.50*** | | | ***0.00*** |
| Your ability to use a vehicle | 38.50 | | 0.50 | | 47.50 | | | 0.59 | | 57.00 | | 0.38 | | | 64.00 | | | 0.22 |
| Your ability to move around within your house | 38.50 | | 0.51 | | 27.50 | | | 0.07 | | 70.00 | | 0.93 | | | 70.00 | | | 0.42 |
| Your ability to move around your neighborhood | 37.00 | | 0.45 | | 26.00 | | | 0.06 | | 60.00 | | 0.50 | | | 59.00 | | | 0.17 |
| Your ability to participate in family activities | 33.00 | | 0.29 | | 28.50 | | | 0.09 | | 60.00 | | 0.50 | | | ***52.50*** | | | ***0.09*** |
| Your ability to assist other members of your family | 33.00 | | 0.29 | | ***17.50*** | | | ***0.02*** | | 59.00 | | 0.46 | | | ***45.00*** | | | ***0.04*** |
| Your ability to participate in community activities | 44.00 | | 0.80 | | 30.50 | | | 0.11 | | 69.00 | | 0.88 | | | 57.00 | | | 0.15 |
| Your ability to participate in activities that you enjoy | 38.50 | | 0.51 | | 30.00 | | | 0.11 | | 64.00 | | 0.65 | | | 61.50 | | | 0.22 |
| Your ability to work | 42.00 | | 0.67 | | 40.00 | | | 0.33 | | 56.50 | | 0.35 | | | ***32.50*** | | | ***0.01*** |
| Your ability to have a close relationship with others | 34.00 | | 0.29 | | ***16.50*** | | | ***0.01*** | | 55.00 | | 0.29 | | | 59.00 | | | 0.17 |
| Your ability to relate to neighbours and friends | 28.50 | | 0.17 | | ***16.50*** | | | ***0.01*** | | ***37.00*** | | ***0.04*** | | | 70.00 | | | 0.43 |
| Your ability to relate to relatives | 35.00 | | 0.36 | | ***17.50*** | | | ***0.01*** | | 49.50 | | 0.19 | | | ***50.00*** | | | ***0.06*** |
| Your ability to relate to people in authority | 34.00 | | 0.32 | | 44.50 | | | 0.49 | | 65.50 | | 0.72 | | | 64.00 | | | 0.27 |
| **Raw Subscale Scores** |  | |  | |  | | |  | |  | |  | | |  | | |  |
| **Psychology** | 37.50 | | 0.47 | | ***2.50*** | | | ***0.00*** | | 71.00 | | 0.98 | | | 50.50 | | | 0.08 |
| **Self Care** | 43.50 | | 0.76 | | 23.00 | | | 0.04 | | 64.50 | | 0.67 | | | ***45.00*** | | | ***0.04*** |
| **Mobility** | 28.50 | | 0.18 | | ***14.00*** | | | ***0.01*** | | 71.00 | | 0.98 | | | ***37.50*** | | | ***0.02*** |
| **Participation** | 44.50 | | 0.83 | | 24.50 | | | 0.06 | | 62.00 | | 0.58 | | | ***44.50*** | | | ***0.04*** |
| **Relationships** | 32.00 | | 0.27 | | 26.00 | | | 0.06 | | 42.00 | | 0.09 | | | 62.00 | | | 0.23 |
|  | | | | | | | Currently Employed | | | | | | | | | | | |
|  | | | | | | | Non UXO | | | | | | | UXO | | | | |
|  | | | | | | | Mann–Whitney | | | Asymp. Sig. | | | | Mann–Whitney | | | Asymp. Sig. | |
| Your overall satisfaction with life | | | | | | | 30.00 | | | 0.08 | | | | 76.00 | | | 0.45 | |
| Your moods and feelings | | | | | | | 39.00 | | | 0.30 | | | | 90.00 | | | 0.96 | |
| Your sense of confidence | | | | | | | 50.00 | | | 0.78 | | | | 54.00 | | | 0.07 | |
| Your ability to live independently | | | | | | | 31.00 | | | 0.11 | | | | 76.00 | | | 0.46 | |
| Your reliance on others for help | | | | | | | 31.00 | | | 0.11 | | | | 79.50 | | | 0.57 | |
| Your ability to wash yourself | | | | | | | 54.00 | | | 1.00 | | | | 63.50 | | | 0.16 | |
| Your ability to use the toilet | | | | | | | 43.00 | | | 0.44 | | | | 73.00 | | | 0.36 | |
| Your ability to dress yourself | | | | | | | 54.00 | | | 1.00 | | | | 73.00 | | | 0.36 | |
| Your ability to feed yourself | | | | | | | 46.00 | | | 0.45 | | | | 85.00 | | | 0.74 | |
| Your ability to sit or stand | | | | | | | 45.50 | | | 0.56 | | | | 71.50 | | | 0.33 | |
| Your ability to carry things | | | | | | | 39.50 | | | 0.32 | | | | 77.50 | | | 0.50 | |
| Your ability to use a vehicle | | | | | | | 33.50 | | | 0.15 | | | | ***50.50*** | | | ***0.02*** | |
| Your ability to move around within your house | | | | | | | 46.00 | | | 0.58 | | | | ***48.00*** | | | ***0.03*** | |
| Your ability to move around your neighborhood | | | | | | | 33.50 | | | 0.16 | | | | ***37.50*** | | | ***0.01*** | |
| Your ability to participate in family activities | | | | | | | 30.00 | | | 0.10 | | | | 54.00 | | | 0.06 | |
| Your ability to assist other members of your family | | | | | | | 36.00 | | | 0.22 | | | | ***51.00*** | | | ***0.05*** | |
| Your ability to participate in community activities | | | | | | | ***17.00*** | | | ***0.01*** | | | | 55.50 | | | 0.07 | |
| Your ability to participate in activities that you enjoy | | | | | | | 36.50 | | | 0.23 | | | | ***52.50*** | | | ***0.05*** | |
| Your ability to work | | | | | | | ***9.50*** | | | ***0.00*** | | | | 59.50 | | | 0.11 | |
| Your ability to have a close relationship with others | | | | | | | 53.00 | | | 0.94 | | | | 70.50 | | | 0.30 | |
| Your ability to relate to neighbours and friends | | | | | | | 53.50 | | | 0.97 | | | | 68.00 | | | 0.24 | |
| Your ability to relate to relatives | | | | | | | 43.00 | | | 0.45 | | | | 66.00 | | | 0.20 | |
| Your ability to relate to people in authority | | | | | | | ***24.50*** | | | ***0.04*** | | | | 67.50 | | | 0.23 | |
| **Raw Subscale Scores** | | | | | | |  | | |  | | | |  | | |  | |
| **Psychology** | | | | | | | 31.00 | | | 0.12 | | | | 72.00 | | | 0.35 | |
| **Self Care** | | | | | | | 51.00 | | | 0.83 | | | | 71.00 | | | 0.32 | |
| **Mobility** | | | | | | | 29.00 | | | 0.09 | | | | ***48.00*** | | | ***0.04*** | |
| **Participation** | | | | | | | ***15.50*** | | | ***0.01*** | | | | 56.50 | | | 0.09 | |
| **Relationships** | | | | | | | 42.50 | | | 0.44 | | | | 61.00 | | | 0.13 | |
|  | | AGE <50 | | | | | | | | | Gender | | | | | | | |
|  | | Non UXO | | | | UXO | | | | | Non UXO | | | | | UXO | | |
|  | | Mann–Whitney | | Asymp. Sig. | | Mann–Whitney | | | Asymp. Sig. | | Mann–Whitney | | Asymp. Sig. | | | Mann–Whitney | | Asymp. Sig. |
| Your overall satisfaction with life | | 53.00 | | 0.25 | | 57.00 | | | 0.11 | | 62.50 | | 0.57 | | | 43.00 | | 0.83 |
| Your moods and feelings | | 70.00 | | 0.93 | | 62.00 | | | 0.18 | | 64.00 | | 0.65 | | | 35.00 | | 0.43 |
| Your sense of confidence | | 62.50 | | 0.59 | | 50.50 | | | 0.06 | | 47.00 | | 0.14 | | | 33.50 | | 0.38 |
| Your ability to live independently | | 70.50 | | 0.95 | | 60.00 | | | 0.16 | | 71.50 | | 1.00 | | | 37.00 | | 0.53 |
| Your reliance on others for help | | 62.00 | | 0.57 | | 56.50 | | | 0.11 | | 67.50 | | 0.81 | | | 38.00 | | 0.58 |
| Your ability to wash yourself | | 61.50 | | 0.51 | | 70.00 | | | 0.35 | | 61.50 | | 0.51 | | | 22.50 | | 0.09 |
| Your ability to use the toilet | | 70.50 | | 0.95 | | 66.50 | | | 0.27 | | 48.50 | | 0.16 | | | 23.00 | | 0.10 |
| Your ability to dress yourself | | 62.50 | | 0.56 | | 54.50 | | | 0.08 | | 60.50 | | 0.47 | | | 21.50 | | 0.08 |
| Your ability to feed yourself | | 67.00 | | 0.71 | | 82.00 | | | 0.74 | | 55.50 | | 0.19 | | | ***16.00*** | | ***0.02*** |
| Your ability to sit or stand | | 61.50 | | 0.55 | | 77.00 | | | 0.58 | | ***37.00*** | | ***0.04*** | | | 31.00 | | 0.30 |
| Your ability to carry things | | 53.00 | | 0.27 | | 64.50 | | | 0.24 | | 63.50 | | 0.63 | | | 27.50 | | 0.20 |
| Your ability to use a vehicle | | 67.00 | | 0.78 | | 85.50 | | | 0.89 | | 49.50 | | 0.18 | | | 35.50 | | 0.41 |
| Your ability to move around within your house | | 61.50 | | 0.55 | | 66.00 | | | 0.25 | | 66.50 | | 0.76 | | | 24.00 | | 0.11 |
| Your ability to move around your neighborhood | | 66.00 | | 0.75 | | 86.50 | | | 0.94 | | 62.50 | | 0.60 | | | 32.50 | | 0.34 |
| Your ability to participate in family activities | | 66.00 | | 0.74 | | ***46.00*** | | | ***0.03*** | | 52.00 | | 0.25 | | | 33.00 | | 0.36 |
| Your ability to assist other members of your family | | 50.50 | | 0.21 | | 53.50 | | | 0.08 | | 60.00 | | 0.50 | | | 33.00 | | 0.36 |
| Your ability to participate in community activities | | 64.50 | | 0.68 | | 56.00 | | | 0.10 | | 52.50 | | 0.26 | | | 41.00 | | 0.72 |
| Your ability to participate in activities that you enjoy | | 66.50 | | 0.76 | | 50.00 | | | 0.05 | | 63.50 | | 0.63 | | | 36.50 | | 0.50 |
| Your ability to work | | 41.00 | | 0.06 | | 66.00 | | | 0.26 | | 61.00 | | 0.51 | | | 41.00 | | 0.72 |
| Your ability to have a close relationship with others | | 68.00 | | 0.82 | | 75.50 | | | 0.52 | | 49.00 | | 0.15 | | | 27.00 | | 0.18 |
| Your ability to relate to neighbours and friends | | ***30.00*** | | ***0.01*** | | 78.00 | | | 0.61 | | 71.00 | | 0.98 | | | 28.50 | | 0.21 |
| Your ability to relate to relatives | | 48.50 | | 0.17 | | 70.50 | | | 0.36 | | 62.50 | | 0.59 | | | 27.00 | | 0.17 |
| Your ability to relate to people in authority | | 57.00 | | 0.38 | | 75.50 | | | 0.52 | | 49.50 | | 0.18 | | | 40.50 | | 0.69 |
| **Raw Subscale Scores** | |  | |  | |  | | |  | |  | |  | | |  | |  |
| **Psychology** | | 67.50 | | 0.82 | | 54.00 | | | 0.09 | | 70.50 | | 0.95 | | | 41.50 | | 0.76 |
| **Self Care** | | 68.00 | | 0.83 | | 63.50 | | | 0.21 | | 49.00 | | 0.17 | | | 22.00 | | 0.09 |
| **Mobility** | | 65.00 | | 0.71 | | 84.50 | | | 0.86 | | 59.50 | | 0.49 | | | 24.50 | | 0.14 |
| **Participation** | | 71.50 | | 1.00 | | 51.50 | | | 0.07 | | 63.00 | | 0.62 | | | 35.50 | | 0.47 |
| **Relationships** | | 43.00 | | 0.10 | | 73.50 | | | 0.46 | | 71.00 | | 0.98 | | | 34.50 | | 0.42 |

Annex 4

**Table 6** **Spearman’s Correlations**

| Gender | Corr |  |  |  |  |  |  |  |  |  |  |  |  |
| --- | --- | --- | --- | --- | --- | --- | --- | --- | --- | --- | --- | --- | --- |
|  | Sig. |  |  |  |  |  |  |  |  |  |  |  |  |
| Age | Corr | 0.12 |  |  |  |  |  |  |  |  |  |  |  |
|  | Sig. | 0.41 |  |  |  |  |  |  |  |  |  |  |  |
| Physical Therapy/Rehab | Corr | 0.01 | 0.13 |  |  |  |  |  |  |  |  |  |  |
|  | Sig. | 0.94 | 0.37 |  |  |  |  |  |  |  |  |  |  |
| Received Prostheses | Corr | -0.05 | -0.19 | 0.00 |  |  |  |  |  |  |  |  |  |
|  | Sig. | 0.72 | 0.21 | 1.00 |  |  |  |  |  |  |  |  |  |
| Currently Employed | Corr | -0.17 | -0.01 | 0.04 | 0.29 |  |  |  |  |  |  |  |  |
|  | Sig. | 0.24 | 0.95 | 0.76 | **0.04** |  |  |  |  |  |  |  |  |
| Pain | Corr | -0.02 | -0.23 | -0.03 | 0.17 | 0.00 |  |  |  |  |  |  |  |
|  | Sig. | 0.89 | 0.11 | 0.85 | 0.25 | 0.99 |  |  |  |  |  |  |  |
| Anxiety | Corr | -0.11 | -0.30 | 0.14 | 0.13 | -0.01 | 0.15 |  |  |  |  |  |  |
|  | Sig. | 0.42 | **0.04** | 0.33 | 0.38 | 0.96 | 0.30 |  |  |  |  |  |  |
| Psychology Raw | Corr | -0.01 | -0.19 | 0.22 | -0.09 | -0.20 | 0.19 | 0.45 |  |  |  |  |  |
|  | Sig. | 0.97 | 0.18 | 0.13 | 0.53 | 0.16 | 0.19 | **0.00** |  |  |  |  |  |
| Self Care Raw | Corr | -0.07 | -0.05 | -0.03 | -0.06 | -0.06 | 0.17 | 0.23 | 0.60 |  |  |  |  |
|  | Sig. | 0.60 | 0.73 | 0.84 | 0.69 | 0.67 | 0.23 | 0.10 | **0.00** |  |  |  |  |
| Mobility Raw | Corr | 0.05 | 0.07 | 0.22 | -0.16 | -0.33 | 0.25 | 0.37 | 0.70 | 0.58 |  |  |  |
|  | Sig. | 0.73 | 0.63 | 0.12 | 0.29 | **0.02** | 0.07 | **0.01** | **0.00** | **0.00** |  |  |  |
| Participation Raw | Corr | 0.13 | -0.22 | 0.06 | -0.17 | -0.44 | 0.23 | 0.18 | 0.64 | 0.43 | 0.73 |  |  |
|  | Sig. | 0.35 | 0.12 | 0.67 | 0.24 | **0.00** | 0.11 | 0.22 | **0.00** | **0.00** | **0.00** |  |  |
| Relationships Raw | Corr | 0.16 | 0.06 | 0.24 | -0.13 | -0.29 | -0.03 | 0.12 | 0.38 | 0.40 | 0.61 | 0.59 |  |
|  | Sig. | 0.25 | 0.67 | 0.09 | 0.39 | **0.04** | 0.86 | 0.39 | **0.01** | **0.00** | **0.00** | **0.00** |  |
|  |  | Gender | Age | Physical Therapy/Rehab | Received Prostheses | Currently Employed | Pain | Anxiety | Psychology Raw | Self Care Raw | Mobility Raw | Participation Raw | Relationships Raw |
| **Correlation is significant at the 0.05 level (2-tailed).** | | | | |  |  |  |  |  |  |  |  |  |
| **Correlation is significant at the 0.01 level (2-tailed).** | | | | |  |  |  |  |  |  |  |  |  |
